# Silencing Relaxin-3 in Nucleus Incertus of Adult Rodents: A Viral Vector-based Approach to Investigate Neuropeptide Function

**DOI:** 10.1371/journal.pone.0042300

**Published:** 2012-08-02

**Authors:** Gabrielle E. Callander, Sherie Ma, Despina E. Ganella, Verena C. Wimmer, Andrew L. Gundlach, Walter G. Thomas, Ross A. D. Bathgate

**Affiliations:** 1 Department of Biochemistry and Molecular Biology, The University of Melbourne, Victoria, Australia; 2 Florey Neuroscience Institutes, The University of Melbourne, Victoria, Australia; 3 Department of Medicine (Austin Health), The University of Melbourne, Victoria, Australia; 4 Department of Anatomy and Neuroscience, The University of Melbourne, Victoria, Australia; 5 School of Biomedical Sciences, University of Queensland, Queensland, Australia; Yale School of Medicine, United States of America

## Abstract

Relaxin-3, the most recently identified member of the relaxin peptide family, is produced by GABAergic projection neurons in the nucleus incertus (NI), in the pontine periventricular gray. Previous studies suggest relaxin-3 is a modulator of stress responses, metabolism, arousal and behavioural activation. Knockout mice and peptide infusions *in vivo* have significantly contributed to understanding the function of this conserved neuropeptide. Yet, a definitive role remains elusive due to discrepancies between models and a propensity to investigate pharmacological effects over endogenous function. To investigate the endogenous function of relaxin-3, we generated a recombinant adeno-associated viral (rAAV) vector expressing microRNA against relaxin-3 and validated its use to knock down relaxin-3 in adult rats. Bilateral stereotaxic infusion of rAAV1/2 EmGFP miR499 into the NI resulted in significant reductions in relaxin-3 expression as demonstrated by ablation of relaxin-3-like immunoreactivity at 3, 6 and 9 weeks and by qRT-PCR at 12 weeks. Neuronal health was unaffected as transduced neurons in all groups retained expression of NeuN and stained for Nissl bodies. Importantly, qRT-PCR confirmed that relaxin-3 receptor expression levels were not altered to compensate for reduced relaxin-3. Behavioural experiments confirmed no detrimental effects on general health or well-being and therefore several behavioural modalities previously associated with relaxin-3 function were investigated. The validation of this viral vector-based model provides a valuable alternative to existing *in vivo* approaches and promotes a shift towards more physiologically relevant investigations of endogenous neuropeptide function.

## Introduction

The relaxin-3 gene was identified in 2002 [1], and is the most recently identified member of the relaxin family of peptides. This family comprises relaxin-1, relaxin-2 and insulin-like peptides 3, 4, 5 and 6, and like all members of the family, relaxin-3 is synthesised as a pre-prohormone and organised in a signal sequence-B-C-A domain configuration. The mature protein is produced by removal of the C chain and the formation of three disulfide bonds between six highly conserved cysteine residues found on the A and B chains [2].

Evolutionary studies revealed that relaxin-3 is under strong purifying selection indicating a highly conserved and important function [3]. Relaxin-3 is produced in the thymus, kidney, spleen, liver, heart and brain of mice [1], but it is the restricted distribution of relaxin-3 in the brain that has attracted most interest. Relaxin-3 is predominantly produced in neurons of the nucleus incertus (NI), a small area in the pontine periventricular gray in mouse [1], [4], [5], rat [6]–[8] and macaque [9], [10], and is also found in an analogous population of neurons in zebrafish [11]. Ascending projections from the NI innervate rostral targets in the mesencephalon, diencephalon and telencephalon, while more modest descending projections innervate regions of the lower brainstem [12], [13]. There are also relaxin-3-expressing neurons in the pontine raphe nucleus, the periaqueductal gray and an area dorsal to the substantia nigra in the rat and mouse brain [5], [7].

Although relaxin-3 can bind and activate relaxin family peptide receptors (RXFP) 1, 3 and 4, its cognate receptor is RXFP3, a G_i_-coupled receptor (GPCR) [14]. The expression pattern of RXFP3 in the brain largely overlaps the distribution of relaxin-3-like immunoreactivity (relaxin-3-LI) in axons and nerve terminals [5], [7] with abundant RXFP3 mRNA and receptor binding sites in olfactory bulb, septum, extended amygdala, ventral hippocampus, cerebral cortex and several hypothalamic and midline thalamic nuclei [7], [15], [16]. Overall, this suggests that RXFP3 is involved in the central processing of sensory signals and supports the possible modulatory role of the relaxin-3/RXFP3 system in stress and anxiety [8], [17]–[19], feeding and metabolism [20]–[24] and arousal and behavioural activation [5], [7], [9], [25].

The majority of relaxin 3-containing neurons in the NI express corticotropin releasing factor (CRF) receptor 1 (CRF-R1) [8], implicating a role in stress. In rats, CRF administration caused elevated c-Fos expression in 65% of relaxin-3 neurons, stress significantly increases relaxin-3 mRNA in the NI and peptide is present in presynaptic vesicles of thalamic and septal nerve terminals suggesting relaxin-3 produced in the NI is released from nerve endings and is involved in regulation of integrated stress responses [8]. Furthermore, repeat forced swim stress increases relaxin-3 mRNA and heteronuclear RNA 30–60 minutes after the second swim, before a return to baseline levels within 2–4 hours [17]. These effects were blunted by pre-treatment with the CRF-R1 antagonist, antalarmin. Interestingly, a principal site of CRF production in the brain is the hypothalamus, which also expresses RXFP3 [7]. This suggests RXFP3 may mediate actions of relaxin-3 on the CRF system, but this remains to be demonstrated experimentally.

RXFP3 modulates neuronal activity in the hippocampus and medial septum (MS) to promote theta rhythm [9], suggesting a role in spatial working memory. Behavioural studies showed that administration of the RXFP3 peptide antagonist Delta (R3(BΔ23–27)R/I5, [26]) into the MS impaired performance in the spontaneous alternation task (SAT) in rats. Delta produced dose-dependent decreases in percentage alternation score (PAS), an effect blocked by co-administration of the RXFP3 peptide agonist R3/I5 [9].

A role for relaxin-3 in energy homeostasis has been suggested on the basis of peptide infusion studies demonstrating effects on body weight, food intake and plasma hormone levels. While a single study reported intracerebrocentricular (ICV) administration of relaxin-3 increased body weight [22], studies from a different group reported no effect on body weight following ICV or intra-hypothalamic nuclei injection of relaxin-3 [20], [21]. However, relaxin-3 administered ICV to rats either in the early light or dark phase significantly but transiently increased food intake [20], an effect mediated by the paraventricular nucleus of the hypothalamus (PVN) [21]. RXFP3-selective pharmacological modulators have also been used to investigate food intake. ICV infusion of R3/I5 increased food intake and this effect was blocked by pre-administration of Delta [26]. Similar findings have been obtained with different RXFP3 agonists and antagonists developed recently [27]. Interestingly, chronic ICV administration of Delta had no effect on feeding, but significantly increased body weight [28].

While the results outlined indicate distinct roles for relaxin-3 in feeding, stress and spatial working memory, there have been inconsistent results from studies of relaxin-3 knockout (KO) mice that are neither consistent between strains nor with results from *in vivo* peptide studies. For example, although one cohort of relaxin-3 KO mice demonstrated lower body weights, a separate cohort housed in a different laboratory demonstrated no genotype-based differences in body weight, but instead displayed a hypoactive phenotype [4], [28]. Both studies were conducted using mice with a mixed background, and genetic differences likely persisted due to independent inbreeding resulting in the fixation of certain strain specific alleles. A more recent study on a separate relaxin-3 KO strain also demonstrated no effects on body weight, although a mild anxiolytic-like phenotype was observed [29]. Backcrossed relaxin-3 KO mice on a C57Bl/6 background demonstrated circadian hypoactivity when provided with access to voluntary running wheels in their home cage [25]. However, no differences in the modalities previously associated with relaxin-3 function including body weight, anxiety-like behavior or responses to stress, were observed. Like many peptide KO mice, lifetime loss of relaxin-3 expression means these models are susceptible to developmental compensation, potentially masking phenotypes of interest.

Despite a variety of neuroanatomical, evolutionary and biochemical investigations and behavioural testing, a definitive role for relaxin-3 remains elusive. As outlined above, KO mice have failed to reproducibly demonstrate a potential endogenous function and much of our current understanding is based on relatively acute peptide infusion studies in rats. Despite the flexibility of this approach there are many limitations including problems of peptide bioavailability. Ultimately, infusion studies fail to investigate endogenous function; rather they demonstrate outcomes of receptor modulation at pharmacological doses in the absence of an appropriate cellular or behavioural context.

This study aimed to develop an alternative approach to investigate endogenous relaxin-3 function in rats, the species in which most of the information on putative roles of relaxin-3 has been collected. We generated a recombinant adeno-associated viral (rAAV) vector expressing microRNA (miRNA) against relaxin-3 to generate adult knockdown rats. The unusually discrete expression profile of relaxin-3 ensured that targeting infusions to the main site of synthesis, the NI, resulted in significant reductions in relaxin-3 expression throughout the brain in the absence of detrimental neuronal or behavioural effects. The long-term stability of this model afforded a preliminary investigation of several behavioural modalities previously associated with relaxin-3 function and the opportunity to confirm that RXFP3 expression levels were not altered to compensate for reduced relaxin-3. The validation of this model provides a valuable alternative to existing *in vivo* models and provides a capacity for more physiologically relevant investigations of endogenous neuropeptide function.

## Results

### Transduction of Relaxin-3 Expressing Neurons of Nucleus Incertus by rAAV1/2 Vectors

**Figure 1:**
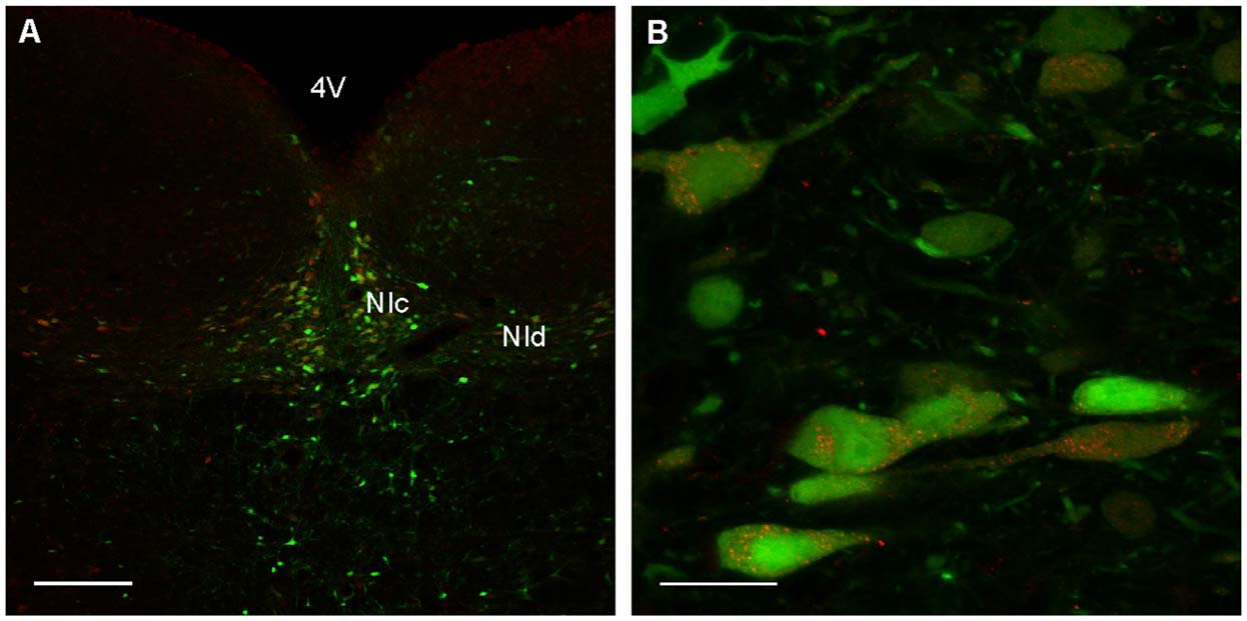
Colocalisation of rAAV1/2 EmGFP and relaxin-3-like immunoreactivity in cells of the nucleus incertus. A. EmGFP transgene expression (green) is colocalised with relaxin-3 immunoreactivity (red) demonstrating that rAAV1/2 EmGFP is capable of transducing the target relaxin-3-containing cells of the nucleus incertus pars compacta (NIc) and nucleus incertus pars dissipata (NId). Scale bar indicates 200 µm. Abbreviation: 4V, 4th ventricle. B. High-magnification image demonstrating cells positive for relaxin-3-like immunoreactivity (red, punctate in the cytoplasm) are transduced by rAAV1/2 EmGFP (green). Scale bar indicates 20 µm.

rAAV with mosaic serotype 1 and 2 (rAAV1/2) capsids have been reported to transduce neurons with greater efficacy than other vectors. To examine whether rAAV1/2 transduced relaxin-3 containing neurons of the NI we made bilateral stereotaxic infusions of 2×10^8^ gc of rAAV1/2 EmGFP vector into the NI of male Sprague Dawley rats (n = 6), which were culled 3 weeks following infusions. Colocalisation of relaxin-3-LI and EmGFP expression demonstrated rAAV1/2 is capable of transducing relaxin-3 expressing neurons of the NIc (nucleus incertus pars compacta) and NId (nucleus incertus pars dissipata)(Figure 1). Neurons of the adjacent dorsal tegmental nucleus remained untransduced, with relaxin-3 neurons being transduced in preference, likely reflecting the absence of receptors responsible for rAAV1/2 receptor-mediated endocytosis on cells of the dorsal tegmental nucleus.

### In vivo Silencing of Relaxin-3 Expression with rAAV1/2 EmGFP miR499

We previously designed three expressed shRNAs (designated miRs) targeting the rat relaxin-3 transcript [30]. *In vitro*, all three miRs significantly reduced relaxin-3 expression, with miR499 (Figure S1) exerting the greatest silencing effect resulting in a 5-fold reduction in expression. Thus, miR499 was adopted for *in vivo* studies.

**Figure 2:**
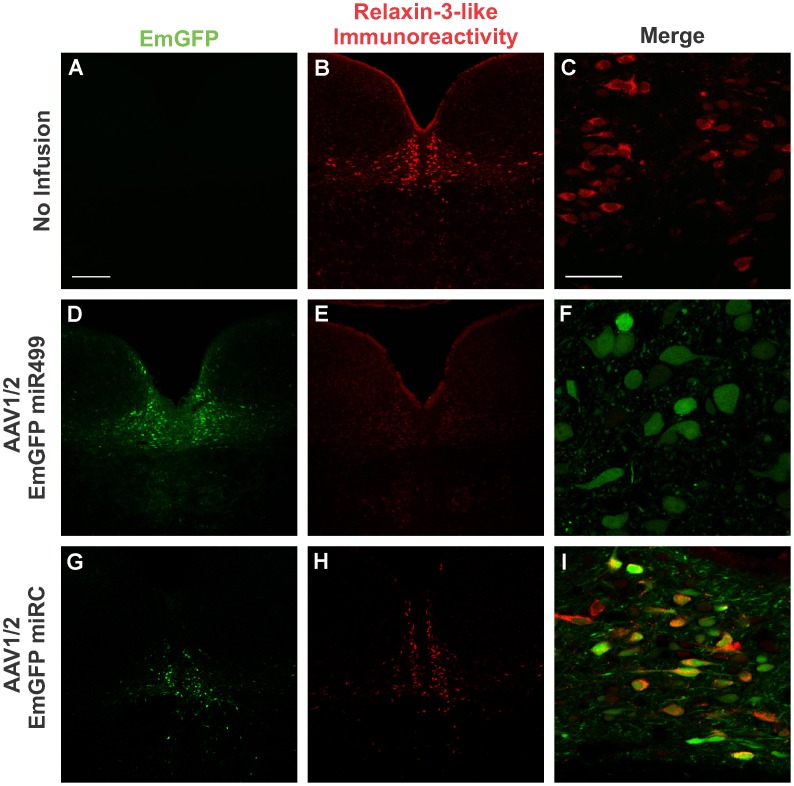
rAAV1/2 EmGFP miR499 reduces relaxin-3-like immunoreactivity in nucleus incertus. EmGFP transgene expression (A, D, G) and relaxin-3-like immunoreactivity (B, E) in no infusion controls (A, B, C) and following bilateral infusion of rAAV1/2 EmGFP miR499 (D, E, F) or rAAV1/2 EmGFP miRC (G, H, I). Scale bar indicates 200 µm (A, B, D, E, G, H) or 50 µm (C, F, I).

The EmGFP miR499 cassette from pcDNA6.2 was cloned into the pAM plasmid, which was packaged into mosaic serotype 1/2 capsids. To test the silencing efficacy of rAAV1/2 EmGFP miR499 *in vivo*, male Sprague Dawley rats received bilateral infusions of rAAV1/2 EmGFP miR499 (6×10^7^ gc, n = 4) into the NI, whereas control rats received either no infusion or rAAV1/2 EmGFP miRC (2.4×10^8^ gc, n = 4). In control rats, high levels of relaxin-3-LI were observed (Figure 2B, C). Three weeks after bilateral infusion of rAAV1/2 EmGFP miR499 into the NI, the EmGFP transgene was robustly expressed, indicating viral transduction and coexpression of miR499 (Figure 2D). Like rAAV1/2 EmGFP, this vector predominantly transduced NI cells, with the nearby dorsal tegmental nucleus remaining free of EmGFP expression. Relaxin-3-LI was absent in the NI of these rats (Figure 2), demonstrating potent silencing of relaxin-3 expression. High-magnification images reveal cytoplasmic EmGFP expression (Figure 2F) without the typical punctate distribution of relaxin-3-LI (Figure 2C).

**Figure 3:**
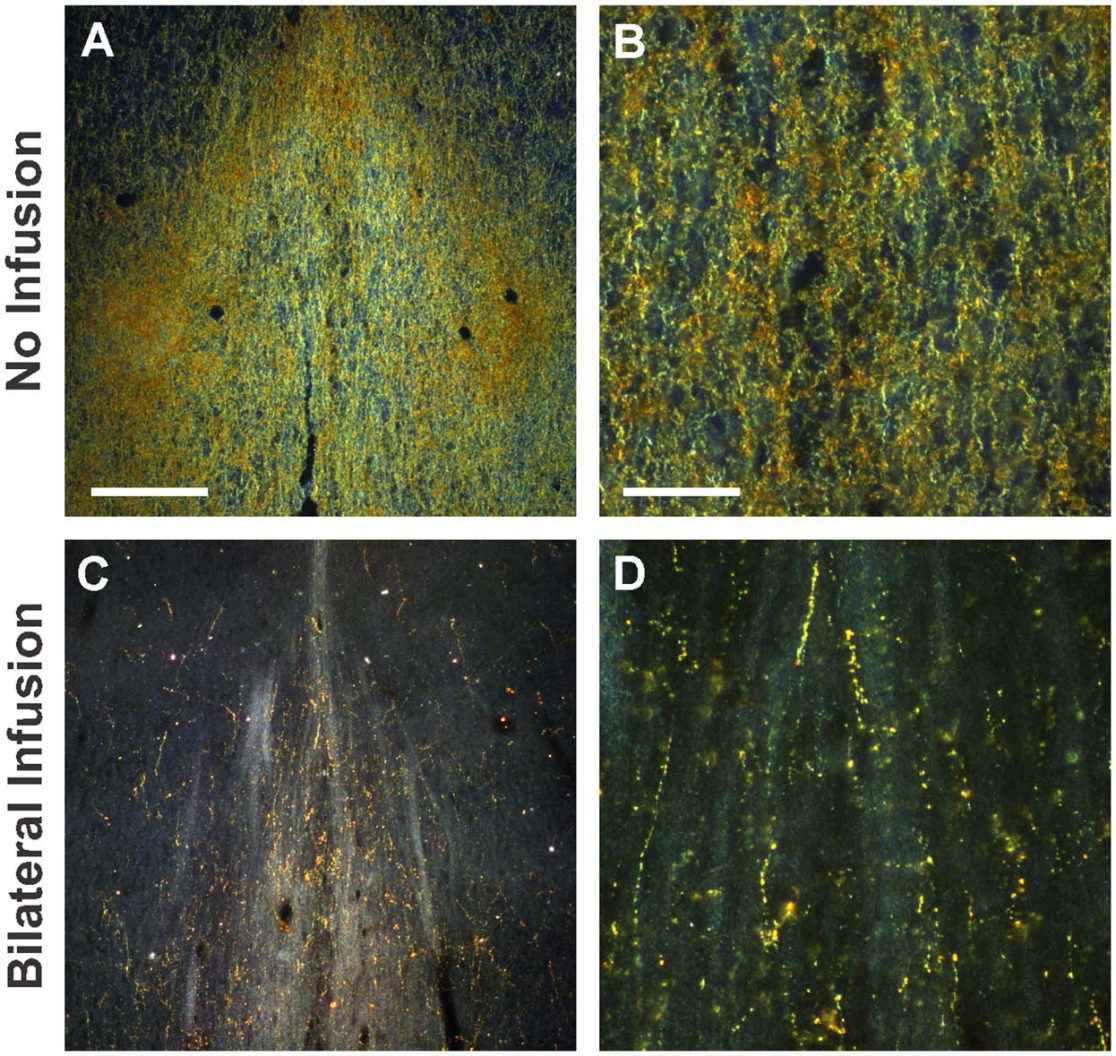
rAAV1/2 EmGFP miR499 reduces relaxin-3-like immunoreactivity in fibres of the septum. A and B received no infusion, n = 4. C and D are 3 weeks following bilateral infusion of rAAV1/2 EmGFP miR499, n = 4. Scale bars indicate 1.5 mm (A, C) and 150 µm (B, D).

Relaxin-3 is present in NI projections and vesicles at nerve terminals, consistent with synaptic release [7], [8]. However, neuropeptide release is not a slow constant event: rather it occurs in bursts in response to a stimulus (see [31] for review). Hence, it is possible that residual relaxin-3 can be translated from the remaining mRNA in transduced neurons and be transported to synapses where it is stored, awaiting release. In this regard, we investigated the relaxin-3-LI in NI projections to the MS. Previous studies have shown relaxin-3-LI in the MS and identified behavioural endpoints related to RXFP3 modulation of this nucleus [9]. Relaxin-3-LI in the MS is visibly reduced 3 weeks after infusion of rAAV1/2 EmGFP miR499 into the NI (n = 4, Figure 3C, D) compared to control levels (n = 4, Figure 3A, B). These results indicate that miR499 induced silencing of relaxin-3, reduces relaxin-3 at both the site of synthesis and in nerve fibres and terminals near the site of relaxin-3 release.

It was important to establish whether reductions in relaxin-3 expression were due to specific gene silencing rather than overall changes in transcription and translation processes or perturbed cellular health. To assess the ability of transduced cells to produce other proteins, expression of the Fox-3 gene, the neuronal-specific nuclear antigen NeuN, was assessed using immunohistochemistry [32]. In rats that received an infusion of rAAV1/2 EmGFP miR499 6 weeks prior (n = 4), there was strong expression of NeuN, in the presence of EmGFP and the absence of relaxin-3-LI (Figure S2A). Nissl stain was used to indicate the state of protein synthesis in transduced neurons. In rAAV1/2 EmGFP miR499 transduced neurons of the NI, high power images demonstrate the Nissl substance was localised in the neuronal soma (Figure S2B). Together, these results suggest neurons transduced by rAAV1/2 EmGFP miR499 are relatively healthy as they are still capable of expressing proteins other than relaxin-3. Therefore, the silencing of relaxin-3 is specific and unlikely to be due to effects on neuronal health or protein expression.

### Time Course of Relaxin-3 Silencing and Transgene Expression Following rAAV1/2 EmGFP miR499 Treatment

**Figure 4:**
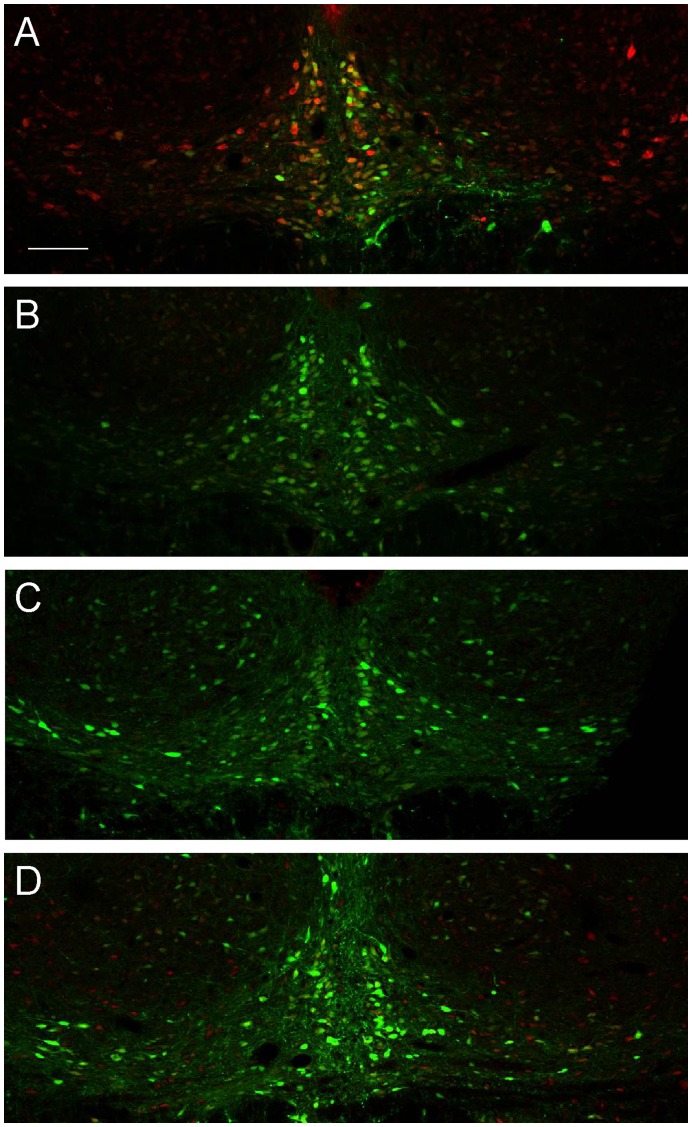
rAAV1/2 EmGFP miR499 reduces relaxin-3-like immunoreactivity from one week post infusion. EmGFP (green) is expressed in cells transduced by rAAV1/2 EmGFP miR499. Relaxin-3-like immunoreactivity (red) in the nucleus incertus decreases as transgene expression increases from 1 week (A), 3 weeks (B), 6 weeks (C) and 9 weeks (D) post infusion. n = 4 rats for each time point. Scale bar indicates 200 µm.

In order to determine the optimal time to begin phenotypic analyses following vector infusion, we investigated the temporal profile of EmGFP transgene expression and relaxin-3 silencing following bilateral infusion of rAAV1/2 EmGFP miR499 (6×10^7^ gc, n = 4 rats per time point). One week following infusion, EmGFP expression was observed in the NI, but relaxin-3-LI was still present in neurons (Figure 4A). As stated, at 3 weeks transgene expression was strong and staining for relaxin-3 was absent (Figure 4B). The same was true for both 6 and 9 weeks following rAAV1/2 EmGFP miR499 infusion (Figure 4C, D). Although all tissue was processed at the same time and under the same conditions, it was not evident whether transgene expression was increased between weeks 3 and 9. It can, however, be stated that relaxin-3-LI was abrogated from week 3 onwards.

### Quantitative Measurement of Relaxin-3 mRNA Following Silencing

**Figure 5:**
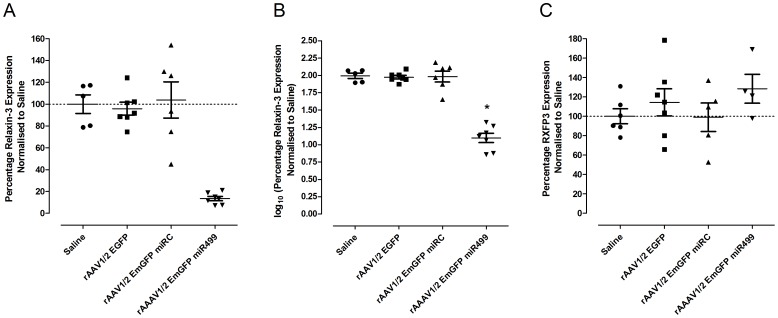
Hindbrain relaxin-3 mRNA is reduced following rAAV1/2 EmGFP miR499 treatment whereas RXFP3 mRNA remains unchanged. qRT- PCR was used to determine the levels in a subset of rats (5–7 rats) for each treatment group. Relaxin-3 levels were reduced compared to all control groups following infusion of rAAV1/2 EmGFP miR499 (A). For statistical analysis relaxin-3 expression was log_10_ transformed (B). *indicates significant difference between the group indicated and all other groups as determine by a one-way ANOVA and Holm-Sidak post-hoc analysis with significance set to p<0.05. There was no significant difference between RXFP3 levels between treatment groups (C).

Whilst immunohistochemistry provided useful information about the stereotaxic infusion and transduction of the NI, the nature of peptide distribution in neurons and technical aspects of the protocol prevented the use of this method for reliable quantitative analysis. Rather, we further confirmed the efficacy of rAAV1/2 EmGFP miR499 by using qRT-PCR to measure relaxin-3 mRNA levels in the hindbrain of a subset of the animals used for behavioural studies (5–7 rats per treatment group). Determination of relative gene expression was performed using the 2^−ΔΔCT^ method as described [33], [34], which included validation of GAPDH as an appropriate internal control gene (Figure S3A, B) and determination of the reaction efficiency for relaxin-3 and GAPDH primers (Figure S3C). The levels of relaxin-3 mRNA for each rat relative to saline infused control animals are displayed in Figure 5A. rAAV1/2 EmGFP miR499 infusion reduced hindbrain relaxin-3 expression to between 7.2 and 21.1% of the average level of the saline infused group, which corresponds to an average expression of 13.4% across the group. Both rAAV1/2 EmGFP and rAAV1/2 EmGFP miRC treatment groups demonstrated greater variability in relaxin-3 mRNA levels than either saline or rAAV1/2 EmGFP miR499 treated groups. Due to this unequal variance, data was normalised for statistical analysis (Figure 5B). A one-way ANOVA indicated that the differences observed between the rAAV treatment groups were significant (F_(3,24)_ = 61.860, p<0.001). Holm-Sidak post-hoc analysis revealed that the rAAV1/2 EmGFP miR499 treated group had significantly less hindbrain relaxin-3 than all other groups (vs EmGFP, t = 11.266, p = 2.31×10^−10^; vs EmGFP miRC, t = 10.899, p = 4.21×10^−10^; vs Saline, t = 10.477, p = 8.52×10^−10^). Additionally, none of the control groups significantly differed in hindbrain relaxin-3 expression.

### Quantitative Measurement of RXFP3 mRNA Following Silencing

One of the major advantages of viral-based animal models over traditional KO and transgenic mice is that the animals are less likely to undergo compensatory changes, as the changes to gene expression do not occur during development. Although this is true, the inherent plasticity of the brain, and the often redundant nature of neuropeptide action, not to mention the numerous receptors that can bind relaxin-3, all contribute to the likelihood of developing compensation in a viral-driven model. If the relaxin-3/RXFP3 system were to compensate for the reduced levels of relaxin-3 following infusion of rAAV1/2 EmGFP miR499, one mechanism could be the up-regulation of RXFP3 expression in nuclei that receive relaxin-3 projections. Consequently, we tested whether administration of rAAV1/2 EmGFP miR499 and relaxin-3 silencing affected RXFP3 mRNA levels in hindbrain mRNA samples from the same subset of rats used for relaxin-3 mRNA quantitation. The determination of RXFP3 primer reaction efficiency was performed as described for relaxin-3 (Figure S3D). Despite substantial and significant reductions in hindbrain relaxin-3 expression, expression of RXFP3 was unchanged in the hindbrain of rAAV1/2 EmGFP miR499 treated animals (Figure 5C). A one-way ANOVA indicated that the differences observed between any of the treatment groups were not significant (F_(3,18)_ = 0.938, p = 0.443), demonstrating that changes in RXFP3 expression were not compensating for reductions in relaxin-3 in this model.

### Measurements of Energy Homeostasis Following rAAV1/2 EmGFP miR499 Treatment

**Figure 6:**
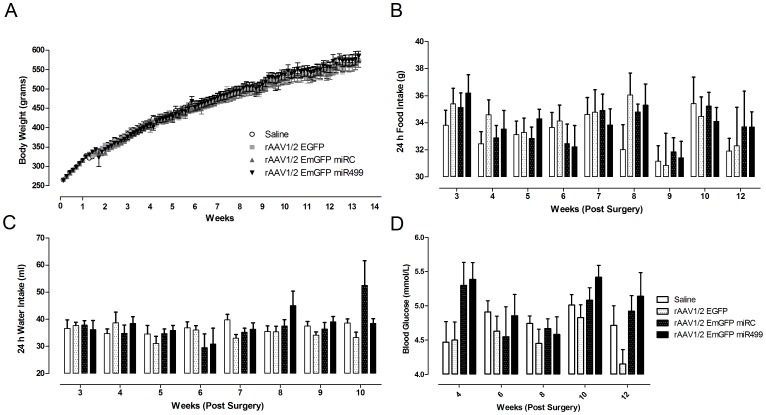
Reduced relaxin-3 does not alter body weight, food or water intake or blood glucose concentration. A. Body weight of each treatment group (mean ± SEM) for the study duration. Stereotaxic surgery was completed between days 7 and 14. B. 24 hr food intake for each treatment group (mean ± SEM) following surgery. C. 24 hr water intake for each treatment group (mean ± SEM) following surgery. Saline n = 13, EmGFP n = 14, miRC n = 13, miR499 n = 14. D. Concentration of blood glucose for each treatment group (mean ± SEM) following surgery. At each time point n = 4–7 for each group.

Next, we examined the phenotype of rats following infusions of rAAV1/2 EmGFP miR499 using metabolic measurements used previously to investigate relaxin-3 function. A role for relaxin-3 in energy homeostasis has been suggested on the basis of peptide infusion studies [20]–[22], [26], whereas studies in KO mice do not corroborate these results [28], [35]. The body weight of each rat was recorded daily as a measure of general health for the duration of the experiment. Following infusions, there were no differences in body weight (Figure 6A, two-way repeated-measures AVOVA F_(3, 432)_ = 0.0706 p = 0.975). This result also included a week in which mash was provided for consumption (days 63–70)). For the subset of rats that underwent qRT-PCR analysis of relaxin-3 expression, it was possible to investigate the relationship between relaxin-3 levels and measured metabolic or behavioural endpoints. There was no significant relationship between relaxin-3 mRNA levels and body weight gain (data not shown, Pearson Product Moment Correlation n = 24, r = 0.0452, p = 0.834).

The effect of reduced relaxin-3 expression on food and water intake was investigated by measuring 24 hr consumption once every seven days. There was no significant difference in the food intake between the control and rAAV1/2 EmGFP miR499 infusion groups at any time point (Figure 6B, two-way repeated-measures ANOVA, F_(3, 451)_ = 0.198, p = 0.897). Similarly, no differences were detected in 24 hr water intake between the groups (Figure 6C, two-way repeated-measures ANOVA, F_(3, 451)_ = 1.412, p = 0.341). In addition, there was no correlation between relaxin-3 expression and food or water intake (data not shown, n = 24, food intake r = −0.0539, p = 0.8026 and water intake r = 0.0346, p = 0.7423).

In an effort to detect any gross abnormalities in metabolism related to food intake, measurements of blood glucose were made in a subset of rats in each group (n = 4–7 at each time point) once a fortnight beginning one week following surgery (Figure 6D). Blood glucose concentrations were between 3.0 mmol/L and 6.6 mmol/L, which are within the normal range (2.7–7.5 mmol/L). A two-way repeated-measures ANOVA indicated that there was an overall effect of rAAV treatment group on blood glucose concentration (F_(3, 123)_ = 3.141, p = 0.043). Holm-Sidak post-hoc analysis revealed that rAAV1/2 EmGFP miR499 had significantly higher blood glucose levels than rAAV1/2 EmGFP treated rats (difference of means = 0.530, t = 2.980, p = 0.007). However, this difference was not significant on individual measurement days. In addition, measurement date had no effect on blood glucose in any treatment group (F_(3, 123)_ = 2.257, p = 0.07).

### Investigation of Rats in Behavioral Paradigms after rAAV1/2 EmGFP miR499 Treatment

**Figure 7:**
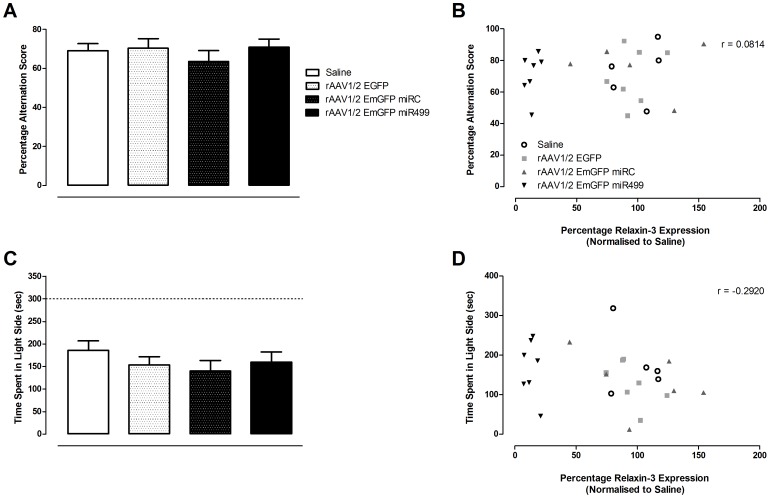
Reduced relaxin-3 has no effect on performance in paradigms of spatial memory or anxiety-like behaviour. A. Spatial learning and memory was assessed in the spontaneous alternation task. Percentage alternation scores for each treatment group are represented as mean ± SEM. Saline n = 13, EmGFP n = 14, miRC n = 13, miR499 n = 14. B. There was no correlation between percentage alternation score and relaxin-3 mRNA levels (n = 24, 5–7 rats per group, r = 0.0814, p = 0.705). C. Anxiety-like behaviour was investigated in the light/dark box. Time spent in the light side for each treatment group is represented as mean ± SEM. Saline n = 13, EmGFP n = 14, miRC n = 13, miR499 n = 14. D. There was no correlation between time and relaxin-3 mRNA levels (n = 24, 5–7 rats per treatment group, r = −0.292, p = 0.166).

Two paradigms were selected for investigation on the basis of behavioural modalities in which relaxin-3/RXFP3 has previously been implicated. Spatial working memory is associated with induction of theta activity in the septohippocampal pathway [9]. Recent studies in our laboratory demonstrated in both anaesthetised and conscious rats, that RXFP3 could modulate neuronal activity in the hippocampus and MS to promote hippocampal theta rhythm [9]. These results were confirmed by behavioural studies demonstrating that blockade of RXFP3 in the MS using Delta resulted in impaired performance in the SAT. Delta produced dose-dependent decreases in PAS, which could be blocked by co-administration of R3/I5. On the basis of these findings, rAAV1/2 EmGFP miR499 infusion and the resultant reductions in relaxin-3 were hypothesised to impair spatial learning and memory. This was investigated using the SAT, a behavioural paradigm commonly used to quantify spatial working memory in rodents [36]–[38]. The PAS of saline infused rats was 69.1% ±3.58% (Figure 7A), which is similar to that reported in previous studies [9]. A higher PAS indicates better alternation and therefore could be interpreted as better spatial memory performance. Infusion of rAAV had no effect on PAS (one-way ANOVA, F(3, 53) = 0.521, p = 0.670). The average number of arm entries made by rats across all treatment groups was not significantly different (data not shown), confirming that the infusions did not affect locomotor ability (one-way ANOVA, F(3, 53) = 0.616, p = 0.608). There was no correlation between relaxin-3 expression and PAS (Figure 5.9C, n = 24, r = 0.0814, p = 0.705).

The septum and hippocampus, along with the amygdala have long been implicated in fear and anxiety. The cholinergic system of the hippocampus and the GABAergic system of the septum act in synergy to modulate anxiety levels [39]. Both these systems have been repeatedly implicated in learning and memory. The MS, amygdala and hippocampus receive strong innervation by fibres of GABAergic relaxin-3 containing neurons in addition to RXFP3 expression being enriched in these regions [7], [16]. Given the role identified for the relaxin-3/RXFP3 system in the MS and the generation of hippocampal theta rhythm, it was important to assess the anxiety of rats following administration of rAAV1/2 EmGFP miR499. Anxiety-like behaviour was assessed following treatment using the well-established unconditioned paradigm, the light-dark box [40]. There were no differences in the time spent in the light side between rAAV treatment groups (Figure 7C, one-way ANOVA, F(3, 46) = 0.791, p = 0.505). Similarly, there was no correlation between relaxin-3 expression and time spent in the light half (Figure 7D, n = 24, r = −0.292, p = 0.166). In addition, rAAV treatment had no effect on the number of entries into the light side (data not shown, one-way ANOVA, F(3, 46) = 1.120, p = 0.269).

In addition to the control measurements taken in the spontaneous alternation task and the light-dark box, locomotor activity was also assessed independently in an automated locomotor cell where activity was monitored during the light phase on three consecutive days for 30 min. Overall, there was no significant effect of rAAV treatment on floor plane distance travelled (data not shown, F(3, 154) = 0.211, p = 0.888), however there was a main effect of test day (data not shown, F(2, 154) = 121.153, p<0.001). There were significant reductions in locomotor activity on the consecutive days of testing, indicating habituation. These results confirm that there were no differences in activity or exploratory behaviour between the groups that could influence performance in the aforementioned paradigms.

## Discussion

Previous studies have suggested a role for the neuronal relaxin-3/RXFP3 system in feeding and metabolism, behavioural activation and arousal and anxiety-like behaviour and the stress response [5], [7]–[9], [17], [20]–[25]. Such studies have utilised either exogenous peptide treatments or a relaxin-3 KO mouse model. Unfortunately, many of the effects of relaxin-3 or RXFP3 pharmacological modulators are unable to be reconciled with the phenotype of the KO mouse. Whether the phenotypes observed in either category of study accurately reflect biological function is not currently clear. Thus, further information is required using these and complimentary experimental approaches.

In this study, we generated an alternative *in vivo* model for the investigation of relaxin-3 function, using a combination of viral gene transfer and gene silencing technology. Infusions of rAAV1/2 EmGFP into the NI demonstrated that mosaic 1/2 particles are capable of transducing relaxin-3 neurons of the NIc and NId. Whilst it was interesting to note, the neurons of the neighbouring dorsal tegmental nucleus remained untransduced, it was not important to restrict transduction to the NI, as the expression of miR499 would be expected to have no effect on neurons that did not produce relaxin-3. Immunohistochemical analysis of relaxin-3 following infusion of rAAV1/2 EmGFP miR499 established that the silencing observed *in vitro* [30] was recapitulated *in vivo*. In several rats, the complete absence of relaxin-3-LI in the NI following a successfully targeted infusion of rAAV1/2 EmGFP miR499 indicated that the levels of relaxin-3 in the NI were substantially reduced. We demonstrated that vector transgene expression was evident from one week following infusion and persisted to the final 12 week time point investigated. Reductions in relaxin-3 were observed as early as one week post-infusion and relaxin-3-LI was entirely absent from 3–9 weeks post-infusion. Finally, using Nissl staining and NeuN immunostaining, we confirmed that vector transduction had no substantial detrimental effects on neuronal health. This data served to reinforce that relaxin-3 is specifically reduced by viral-driven miRNA, rather than a non-specific mechanism, such as overall reductions in protein expression, saturation of protein synthesis machinery, or cell death.

To confirm these results, qRT-PCR was used to measure relaxin-3 mRNA in the entire hindbrain of rats that received rAAV1/2 EmGFP miR499 or a control infusion. Importantly, this provided a quantitative measure for relaxin-3 expression in all treatment groups, which built upon the spatiotemporal information of transgene expression and qualitative measures of relaxin-3 KD. The average relaxin-3 expression in rats of the rAAV1/2 EmGFP miR499 treatment group was only 13% of the equivalent saline infusion control group, verifying the KD already observed using immunohistochemistry. Therefore, measurements at both the level of mRNA and peptide confirm that rats that receive rAAV1/2 EmGFP miR499 do indeed have reduced levels of relaxin-3 compared to their control counterparts. Success in using rAAV1/2 EmGFP miR499 treatment to produce adult rats with reduced relaxin-3 enabled examination of endogenous relaxin-3 function.

As part of the validation of this novel rodent model, several behaviours and associated paradigms used previously to investigate relaxin-3 function were utilised. It is important to note, the aim was not to simply reproduce the phenotypes of previous models in a new model. Rather, having demonstrated that we have effectively decreased NI relaxin-3 expression, we aimed to test existing hypotheses about relaxin-3 function. We investigated the effects of reduced relaxin-3 on body weight, food and water intake, plasma glucose and paradigms related to locomotor activity, spatial learning and memory and anxiety. Behavioural testing did not reveal any phenotypic differences between rats that received rAAV1/2 EmGFP miR499 compared to control infusions. It was initially thought that analysing data from rats based simply on their treatments may have influenced the results, as not all rats in the rAAV1/2 EmGFP miR499 group would have identical KD. However, qRT-PCR analysis of relaxin-3 mRNA levels in a subgroup of rats allowed reanalysis of the behavioural data relative to relaxin-3 expression. In this subgroup, there was a lack of correlation between relaxin-3 mRNA and behaviour in any paradigm used to assess energy balance, locomotor activity, spatial working memory or anxiety.

Although data from mRNA and peptide measurements indicate that relaxin-3 levels are reduced in the rAAV1/2 EmGFP miR499 rats, there is some discrepancy as to the magnitude of the changes. To remove any doubt as to the efficacy of the approach, it was important to establish that relaxin-3 peptide is reduced at its sites of action. Previously, acute infusions of the RXFP3 antagonist Delta into the MS of rats were shown to impair spatial learning and memory [9], but, in this chronic paradigm relaxin-3 KD had no effect. We postulated that in spite of mRNA reductions, retention of relaxin-3 protein in the MS could account for the absence of a spatial learning and memory deficit. However, in rats that received NI infusions of rAAV1/2 EmGFP miR499, the density of relaxin-3-LI fibres in the MS is markedly reduced. Thus, we could conclude that relaxin-3 peptide levels were indeed reduced at sites distal to the NI and the absence of a spatial memory deficit was not due to poor KD. Instead it may be related to the chronic nature of the KD treatment or the elimination of relaxin-3 peptide in all targets of the NI, which include several nodes of the septohippocampal pathway (including the median raphe, supramammillary nucleus and the hippocampus itself), rather than acute blockade of RXFP3 signalling only in the MS.

The residual relaxin-3 mRNA in the hindbrain and the relaxin-3-LI of the septum evokes a common question faced by those attempting gene silencing studies: was gene expression sufficiently impaired to observe a phenotype? In contrast to relaxin-3 KO mice, the rats in this study were still capable of producing relaxin-3, albeit at substantially lower levels. The remaining relaxin-3 present in NI nerve terminals may have been sufficient to confer a functional effect. For example, if the relaxin-3/RXFP3 system were to compensate for the reduced levels of relaxin-3 following infusion of rAAV1/2 EmGFP miR499 by up-regulating expression of RXFP3 in nuclei that receive relaxin-3 projections, function could be maintained. KO mice may also demonstrate increased RXFP3 expression when compared to their wild-type counterparts, but in the absence of relaxin-3, compensation in this manner would go unnoticed. Thus, we examined whether RXFP3 expression is increased as a consequence of relaxin-3 KD. Indeed, as determined by qRT-PCR, hindbrain RXFP3 mRNA levels were not significantly increased in rats that received rAAV1/2 EmGFP miR499 compared to controls. However, to entirely exclude the possibility of nuclei-specific upregulation of RXFP3, it would be necessary to examine RXFP3 expression in forebrain nuclei related to the behaviours investigated. In addition to the residual relaxin-3 produced by the NI, it is also possible that relaxin-3 produced outside the NI is influencing behaviours measured in this study. In addition to the residual relaxin-3 produced by the NI, it is also possible that relaxin-3 produced outside the NI is influencing behaviours measured in this study. Tanaka and colleagues have shown that although the NI has the greatest number of relaxin-3 expressing neurons [8], there are also sparse populations of relaxin-3 expressing neurons in the pontine raphe nucleus, an area dorsal to the substantia nigra and the periaqueductal grey [5], [7].

More generally, neuropeptides demonstrate a high degree of plasticity. Several peptides are preferentially released when neurons are strongly activated, challenged or under pathological conditions [41]. This implies that KD may only have a dramatic effect on a neuropeptide system under conditions where endogenous neuropeptide release (tone) is typically high. Although yet to be demonstrated using RNAi, the importance of increased neuronal excitability and synaptic plasticity has been demonstrated in studies utilising receptor antagonists to investigate neuropeptide function. For example, Chauveau and colleagues recently observed that acute immobilisation stress was required prior to intra-amygdala administration of the neuropeptide S receptor antagonist, SHA68, to produce a demonstrable effect on conditioned fear [42]. Similarly, the κ opioid receptor antagonist, nor-binaltorphimine, and prodynorphin gene disruption were used to block stress-induced responses in behavioural paradigms of addiction [43]. Interestingly, nor-binaltorphimine had no effect on the cocaine conditioned place preference in the absence of stress and similarly, the effect of prodynorphin gene disruption on cocaine conditioned place preference was only observed in swim stressed rats. Such studies highlight the importance of conducting behavioural testing in high tone situations.

Therefore, knowledge about the synthesis of relaxin-3 and stimuli for release is important in order to stimulate relaxin-3 release in behavioural paradigms, ensuring the best opportunity to observe a phenotype. Neurons of the NI are activated by a variety of stimuli, however only two studies have also extended their investigation to measuring relaxin-3 expression following stimulus and NI activation. Tanaka and colleagues demonstrated that following persistent water restraint stress NI c-Fos and relaxin-3 mRNA levels were increased [8] and we demonstrated that repeat forced swim could increase relaxin-3 mRNA levels in the NI of rat [17]. Thus, our current understanding of the stimuli responsible for relaxin-3 expression leads to the hypothesis that although relaxin-3 mRNA and peptide is reduced following administration of rAAV1/2 EmGFP miR499, in the absence of stimuli for relaxin-3 release, the residual peptide might accumulate in the neuron and when release occurs, levels are not substantially impaired. According to Hökfelt and colleagues, stimuli such as stress, learning and fear result in high or bursting neuronal activity [31]. The role of relaxin-3 in any conditions that would induce what is considered high, maximal or excessive neuronal activity has not yet been examined. As the relaxin-3/RXFP3 network is associated with pathways related to reward and 5-HT depletion increases relaxin-3 expression [44], animal models of narcotic effects and addiction, may demonstrate altered relaxin-3 release or RXFP3 expression. The most extreme scenario of peptide release, the diseased state, may also reveal alterations in relaxin-3 synthesis and release; however it would be important to focus on diseases in which brain areas that express relaxin-3 or RXFP3 are affected.

On the basis of these theories, we propose the absence of a phenotype in relaxin-3 KD rats is primarily due to a lack of high or bursting neuronal activity during behavioural testing. Future studies utilising the relaxin-3 KD rat could account for endogenous relaxin-3 tone by either pre-stressing animals prior to behavioural testing or alternatively, testing animals in behavioural paradigms that in themselves result in excessive levels of neuronal activation and neuropeptide release (e.g. the auditory fear-conditioning paradigm and models of addictive behaviour such as cue-induced reinstatement).

In summary, we have demonstrated the use of viral vector-driven silencing of relaxin-3 to generate an adult rat model to investigate endogenous relaxin-3 function. Significant reductions in relaxin-3 expression were achieved, in the absence of detrimental neuronal or behavioural effects. No behavioural phenotype was detected in the paradigms tested. Behavioural testing following KD was conducted under conditions typically associated with low neuropeptide tone, which we propose may yield less dramatic functional consequences than high tone scenarios. Future experiments will focus on the conditions of relaxin-3 synthesis and release and whether NI activity and associated relaxin-3 tone influences the development of behavioural phenotypes.

## Materials and Methods

### Cloning

The rAAV plasmids pAM-DCA-EcoRI-EGFP (pAM), pDPI and pDPII were provided by Jűrgen Kleinschmidt (Heidelberg, Germany) [45]. EmGFP miR sequences were amplified using PCR from the pcDNA6.2-GW/EmGFP miR vectors previously described [30] using the forward primer (5′- CAT CAT CAT GAA TTC CAA GCT GGC AAG TTA-3′) and the reverse primer (5′-CAT CAT CAT AAG CTT TCT AGA TAT CTC GAG TGC GG-3′) and cloned into pAM. Final plasmids were digested with SmaI to ensure the inverted terminal repeats were intact and both strands of four clones for miR499 and miRC were sequenced using forward primer (5′-GCT CCT GGG CAA CGT GC-3′) and reverse primer (5′-GCC ATA CGG GAA CGA ATA GC-3′).

### rAAV Production

AAV 293 cells (Stratagene, Singapore) were grown in Dulbecco’s Modified Eagles Medium (DMEM, Gibco-BRL, Mulgrave, Australia ) supplemented with 10% fetal bovine serum (Gibco-BRL), 100× non-essential amino acids (Gibco-BRL), 100× sodium pyruvate (Gibco-BRL) and 1% penicillin/streptomycin (Gibco-BRL) at 37°C in a humidified incubator with 5% CO_2_. Cells were passaged as required to maintain confluency below 50%. For transfection, cells were always passaged the day prior and used between passage 7 and 30 at 60% confluency. rAAV particles were produced following a previously described calcium phosphate transfection protocol [46]. For preparative large-scale productions, 1.5×10^7^ AAV 293 cells were seeded in each of ten 15 cm culture dishes. Cells were transfected using the calcium phosphate method with 50 µg DNA consisting of equimolar amounts of pAM-EmGFP (vector, miRC or miR499) and the two helper plasmids, pDPI and pDPII [45]. Media was replaced after 16 hr and 60–72 hr following transfection, cells and media were harvested and pelleted. The supernatant was removed and the pellet resuspended in lysis buffer (150 mM NaCl, 50 mM Tris-HCl pH 8.5). The cell suspension was then subjected to four freeze-thaw cycles and treatment with benzonase endonuclease (50 U/ml, Sigma, Sydney, Australia). The lysate was clarified by centrifugation and the vector containing supernatant was filtered through a 13 mm diameter 45 µm filter (Millipore, Kilsyth, Australia) to yield a crude lysate.

Vector particles were purified and concentrated using discontinuous iodixanol gradient centrifugation [47]. A 60% (w/v) sterile solution of OptiPrep (Sigma) was used to make 15%, 25%, 40% and 54% (w/v) iodixanol solutions. In heat-sealable centrifuge tubes (Hitachi, Japan), the discontinuous step gradient was formed by underlayering and displacing the less dense crude viral lysate with increasingly dense iodixanol solutions. Gradients were centrifuged at 350,000 g_av_ for 1 hr at 18°C. The 40% fraction was extracted at the interface between the 40/54% layers, concentrated in an Amicon centricon filter device (Millipore) and dialysed with PBS. A final volume of 250–500 µl was collected and filtered through a 13 mm 22 µm filter unit (Millipore) and stored at 4°C in preparation for titration and use. For long term storage, small aliquots were made and vectors stored at −80°C. Once thawed for use, vector was never re-frozen and was stored at 4°C.

The vector titres were determined using a qPCR-based protocol adapted from Veldwijk and colleagues [48]. The DNA plasmid pAM-DCA-EcoRI-EGFP was used for the qPCR standard, the concentration of which was measured using absorbance at 260 nm (Nanodrop ND-1000 Spectrophotometer, Nanodrop, Wilmington, DE, USA). Reactions were carried out in a total volume of 10 µl containing 2 µl of 1x SYBR Green JumpStart Taq Ready Mix (Sigma), 2 µl (1 µM) GFP primers (forward 5′-CTG ACC CTG AAG TTC ATC TGC ACC AC-3′, reverse 5′-TCC AGC AGG ACC ATG TGA TC-3′), 4 µl of H_2_0 and 2 µl of either sample or standard. qPCR amplification was carried out on an PE-ABI Prism 7700 Sequence Detection System version 1.9.1 (Applied Biosystems, Mulgrave, Australia) with the conditions 2 min at 50°C, 10 min at 95°C (polymerase activation/pre-denaturation), 40 cycles of denaturation at 95°C for 15 sec and annealing/extension at 60°C for 1 min, with a denaturation cycle at the end of 95°C for 15 sec, 60°C for 1 min and 95°C for 15 sec. Amplification of PCR products of the correct length was confirmed by running PCR products on a 1% agarose gel.

Indications of infectious titre were obtained by transducing HT1080 cells and visualizing GFP expression 4 days later. HT1080 cells were maintained in DMEM supplemented with 10% FBS, 1% penicillin/streptomycin and 1% L-glutamine at 37°C with 5% CO_2_ and 85% humidity in a CO_2_ water-jacket incubator. Cells (80,000/well) were seeded in a 24-well plate containing coverslips coated with 0.01 mg/ml poly-L-lysine (Sigma). The following day cells were transduced with a series of dilutions of vector in PBS: 1 µl purified vector, 1∶10, 1∶100, 1∶1,000, 1∶10,000 and mock. Two days later, media was changed. A further two days later, cells were washed once with warm PBS and fixed in ice cold 4% PFA in the dark for 10 min. Cells were washed again with PBS and coverslips were mounted onto gelatin-coated slides using Dako fluorescent mounting medium (Dako, Campbellfield, Australia). Transduction was assessed on an Olympus IX fluorescence microscope (Olympus, Mt Waverley, Australia).

### Animals

Experiments described were conducted with the approval of the Florey Neuroscience Institutes Animal Ethics Committee and according to ethical guidelines issued by the National Health and Medical Research Council of Australia. All efforts were made to minimize the number of rats used. Male Sprague-Dawley rats were obtained from The Animal Resources Centre (Canning Vale, WA, Australia). They were group-housed prior to surgery in a temperature and humidity controlled room (21±2°C) under a 12 hr light/dark cycle initiated at 0700 hr with *ad libitum* access to standard pelleted food and water unless otherwise stated.

### Surgical Procedures

Rats (250–270 g) were anaesthetised by inhalation of 4% isoflurane delivered in air (1 L/min) and the head was positioned in a stereotaxic frame (Kopf Instruments, Tujunga, CA, USA) using ear and incisor bars. Anaesthesia was maintained with 1–2% isoflurane delivered at 200 ml/min in air using a rat inhalation mask (Kopf Instruments). Bilateral infusions of 2 µl rAAV (rAAV1/2 EmGFP 1.1×10^8^ gc/µl, rAAV1/2 EmGFP miRC 4.5×10^7^ gc/µl, rAAV1/2 EmGFP miR499 5×10^7^ gc/µl) or saline were made into the NI (AP −9.5 mm, ML±0.5 mm, DV −7.6 mm from bregma according the rat stereotaxic atlas [49]). Injections were made at a rate of 0.5 µl/min via a 30 G needle connected to a 10 µl Hamilton Syringe mounted on an infusion pump (Harvard Apparatus, Holliston, MA, USA). Following the infusion, the needle was kept in place for 1 min, retracted 1 mm, kept in place for a further 5 min before complete withdrawal to maximise viral diffusion. After surgery, each rat received an injection of meloxicam (3 mg/kg, subcutaneously). The incision was sutured and the rats were allowed to recover for 7 days.

### Behavioural Testing

Four treatment groups were used in behavioural and biochemical studies; saline (n = 13), rAAV1/2 EmGFP (n = 14), rAAV1/2 EmGFP miRC (n = 13) and rAAV1/2 EmGFP miR499 (n = 14). Following behavioural testing rats were subdivided into two categories, those for which a quantitative measure of relaxin-3 expression (qRT-PCR) was made and those for which immunostaining was conducted. For the former, correlations between relaxin-3 expression and behaviour were made, whereas for the latter, general statements about the behaviour observed by a treatment group were made.

### Body Weight, Food and Water Intake

Body weight was measured and recorded during the light phase daily between 0930 and 1130 hrs. Measurements of food and water consumption were made once every 7 days in the home cage. Pre-weighed food (100 g) was provided following regular daily body weight measurements and 24 hr later, the remaining food was removed (including any uneaten food in the cage) and weighed. Water measurements to the nearest ml were made by weighing the water bottle at the beginning and end of the 24 hr period.

### Blood Glucose

Blood was collected using the tail clip method once every 2 weeks, beginning 2 weeks after stereotaxic surgery. Blood was collected after 1130 hr following the routine daily weighing. Glucose concentrations were measured using MediSense TrueSense Blood Glucose meter and electrodes (Abbott Laboratories, Lane Cove, Australia).

### Locomotor Activity

Locomotor activity of rats in a 30 min period was recorded between 1000 hr and 1600 hr on 3 consecutive days. Rats were placed in a 41 cm ×41 cm ×41 cm clear walled locomotor cell (Tru Scan Photobeam Arena, E63-20; Coulbourn Instruments, Whitehall, PA, USA), with an array of photobeam detectors spaced 2.54 cm apart to track horizontal and vertical movements (rearing events) to within 1.27 cm. Tru Scan 2.0 software (Coulbourn Instruments) automatically determined the total distance travelled, number of moves and number of vertical plane entries.

### Spontaneous Alternation Task

The SAT was conducted in a closed-arm plus maze as described [38]. The arms were 75 cm long, 10 cm wide and had walls 17 cm high. Between testing each animal, the maze was cleaned with 30% ethanol, rather than 70% to prevent changes in behaviour due to the aversive scent of ethanol. Spatial cues were minimised in the testing room. Rats were allowed at least 20 min to become accustomed to the room prior to testing. Rats were placed in the centre of the closed-arm maze, always facing the same direction and allowed 10 min of unimpeded exploration. Activity was recorded by a video camera fixed to the ceiling of the room. The number and sequence of arm entries were manually recorded and used to calculate the PAS. An alternation consisted of four different arm entries in five consecutive arm entries. PAS was calculated by dividing the number of observed alternations in overlapping quintuplets by the number of possible alternations (total number of arms entries −4) and multiplying the result by 100 [50], [51].

### Light/Dark Box

Using the same 41 cm ×41 cm ×41 cm clear-walled locomotor cell (Tru Scan Photobeam Arena, E63-20; Coulbourn Instruments) described for locomotor activity, one half of the cell was defined as ‘dark’, while the other side was defined as ‘light’. This was achieved by placing a box (20.5 cm ×41 cm ×41 cm) in one side of the cell, which was made of a plastic that is impermeable to visible light, but not the locomotor cell photobeams (Light-Dark Box, E63-26, Coulbourn Instruments). The light side was lit by an array of light emitting diodes (500 lux in the centre of the light side, 450 lux in the corners), creating an aversive arena/stimulus. A small hole (7×7 cm) was present in the bottom centre of the dark box to allow rats free passage between the light and dark sides. Trials of 10 min were used.

### Tissue Collection

For histology, rats were culled by isoflurane inhalation overdose and transcardially perfused with 300 ml ice-cold PBS solution (137 mM NaCl, 2.7 mM KCl, 11.2 mM Na_2_HPO_4_, 1.8 mM KH_2_PO_4_, pH 7.4) followed by 400 ml ice-cold 4% paraformaldehyde in PBS (pH 7.4). Rats were decapitated and brains were dissected and immersed in fixative for 1 hr at 4°C before cryoprotection in 30% sucrose in PBS overnight at 4°C. Sections were then snap-frozen over liquid nitrogen, coated in OCT embedding medium (Tissue-Tek, Kirwin, Australia) and stored at −80°C. Series of coronal sections (40 µm) through the NI were cut on a cryostat at −16°C (Cryocut 1800, Leica Microsystems, North Ryde, Australia) and collected free-floating into PBS immediately before immunohistochemical processing.

For the collection of fresh tissue for RNA extraction rats were culled by isoflurane inhalation overdose and decapitation. All surgical equipment was cooled to 4°C and cleaned in 100% ethanol, dried and rinsed in DEPC-treated water prior to use. Brains were rapidly removed and divided into forebrain and hindbrain portions by placement in a brain block at 4°C, ventral surface up and cutting in the coronal plane between the pons and hypothalamus. Brain tissue was then snap frozen by suspension over liquid nitrogen and stored at −80°C until use.

### Relaxin-3 and RXFP3 qRT-PCR

Total RNA was extracted using RNeasy Midi kits (Qiagen, Doncaster, Australia) according to the manufacturer’s instructions. For each sample, 1 µg of total RNA was reverse transcribed into cDNA using TaqMan Reverse Transcription reagents (Applied Biosystems) with random hexamers. Reverse transcription reactions were performed on a PCR Thermal Cycler Dice (Takara, Cheltenham, Australia) with the following conditions 25°C for 10 min, 42°C for 30 min, 95°C for 5 min and hold at 4°C. The cDNA products were stored at −20°C for subsequent use.

All gene expression studies were conducted using qPCR performed on the PE-ABI Prism 7700 Sequence Detection System version 1.9.1 (Applied Biosystems). Reactions were performed in 96 well plates and run using standard conditions; 2 min at 50°C, 10 min at 95°C and 40 cycles of 15 sec at 95°C and 1 min at 60°C with fluorescence measured after each cycle. Data was analysed using the 7500 Fast System Sequence Detection Software (Applied Biosystems).

Three rodent genes were assessed for suitability for use as an internal control gene [34], 18S (M11188), βactin (NM_031144) and GAPDH (NM_017008). Validation of each potential control was required to determine if rAAV treatment had an effect on expression. Primers were designed against the cDNA sequences of each using Primer Express Software for Real-Time PCR 3.0 (Applied Biosystems). Primer pairs selected for each were as follows:

18S forward5′-TCGGAACTGAGGCCATGATT-3′

18S reverse5′-TTTCGCTCTGGTCCGTCTTG-3′

βactin forward5′-CTAAGGCCAACCGTGAAAAGAT-3′

βactin reverse5′-AGAGGCATACAGGGACAACACA-3′

GAPDH forward5′-CTACCCCCAATGTATCCGTTG-3′

GAPDH reverse5′-AGCCCAGGATGCCCTTTAGT-3′

For each candidate gene, four samples were analysed from each treatment group; saline, rAAV1/2 EmGFP, rAAV1/2 EmGFP miRC and rAAV1/2 EmGFP miR499. 5 µL cDNA was added to wells followed by master mix containing 10 µL SYBR Green JumpStart Taq ReadyMix (Sigma), 20 µM forward and reverse primers and made up to volume with injection water.

Statistical analysis was performed using SigmaStat Version 3.5 (Systat Software, Chicago, IL, USA) unless otherwise stated. For candidate internal control genes, a two-way repeated measures ANOVA was used to analyse the average 2^−CT^ values from four brains in each treatment group (saline, EmGFP, miRC, miR499). When miRNA treatment was found to have a significant effect on average 2^−CT^ values, Holm-Sidak multiple comparisons versus saline control post-hoc analysis with significance set to p<0.05 was used to identify which treatment group was affected. The gene that showed the smallest effect between groups was chosen as the internal control.

Determination of relative gene expression of relaxin-3 and RXFP3 was performed using the 2^−ΔΔC^T method as described [33], [34]. The primers were as follows:

Relaxin-3 forward5′-CTACCCCCAATGTATCCGTTGT-3′

Relaxin-3 reverse5′-TAGCCCAGGATGCCCTTTAGT-3′

RXFP3 forward5′-GCAGCCCCCATGAGTAAGG -3′

RXFP3 reverse5′-ACGCATTGCTGCTCCTGTTG -3′

Briefly, qPCR was performed on 5–7 samples in the 4 rAAV treatment groups in triplicate for both relaxin-3, RXFP3 and GAPDH. 5 µL cDNA was added to wells followed by master mix containing 10 µL SYBR Green JumpStart Taq ReadyMix, forward and reverse primers and made up to 20 µL volume with injection water. On the basis of primer optimisation and amplification efficiency studies, primer concentrations were different for GAPDH and relaxin-3. For GAPDH 1 µl of 20 µM forward primer and 1 µl of 20 µM reverse primer were used. For relaxin-3, 0.5 µl of 20 µM forward primer and 1 µl of 20 µM reverse primer were used. Using SigmaStat, a one-way ANOVA with Holm-Sidak post-test was used to determine the effect of treatment on relaxin-3 expression.

### Immunohistochemistry

Free-floating sections were incubated in blocking buffer (0.05 M Tris-buffered saline containing 0.3% (v/v) Triton X-100 (TBX), 10% (v/v) normal horse serum (NHS) and 2% BSA) for 1 hr with shaking at room temperature. Sections were then incubated in TBX containing 1∶1000 rabbit polyclonal anti-relaxin-3 [7], 2% NHS and 2% BSA for 16 hr at 4°C. Sections were washed 3×10 min in TBX and then incubated in 1∶500 Alexa549-conjugated goat anti-rabbit IgG (Invitrogen, Mulgrave, Australia) in TBX for 3 hr at room temperature. Sections were then washed 3×10 min in Tris-buffered saline and mounted on gelatin-chrom alum-coated glass microscope slides and cover-slipped with 2–3 drops of fluorescent mounting medium (Dako).

For relaxin-3 and NeuN (Neuronal Nuclei antigen) double immuno-labelling a 1∶1000 dilution of mouse monoclonal NeuN antibody (Chemicon, Boronia, Australia) added to the anti-relaxin-3 primary incubation. For detection of the NeuN antibody, a 1∶500 dilution of Alexa405-conjugated goat anti-mouse IgG (Invitrogen) was added to the secondary antibody incubation.

### Nissl Staining

Coronal sections were mounted on gelatin-chrom alum-coated glass microscope slides and left to dry in the dark for 72 hr. Slide mounted sections were rehydrated for 40 min in 0.1 M PBS and then permeabilised in PBS containing 0.1% Triton X-100 for 10 min at room temperature. Slides were then washed 2×5 min in PBS. A 1∶200 dilution of NeuroTrace 530/615 red fluorescent Nissl stain (Invitrogen) in 0.1 M PBS was added and incubated in the dark at room temperature for 20 min. The stain was then removed by washing in 0.1 M PBS containing 0.1% Triton X-100 for 10 min followed by 2×5 min washes in 0.1 M PBS. Slides were coverslipped with 2–3 drops of fluorescent mounting medium.

### Microscopy

A confocal microscope with 405/473/559 diode lasers (Olympus IX81 inverted FV 1000 microscope) was used to obtain images of all fluorescent immunohistochemistry. Either a UPLSAPO 10×0.40 numerical aperture water immersion lens or UPLSAPO 60×1.35 numerical aperture oil immersion lens was used. Images of EmGFP were taken by using an excitation wavelength of 473 nm with emission collected at 510 nm. For simultaneous imaging of Alexa594 (relaxin-3-LI), an additional channel (excitation 559 nm and emission 618 nm) was used. Similarly, excitation at 559 nm was used for the detection of NeuroTrace red Nissl stain, with an emission of 615 nm. Alexa405 was detected using an excitation of 405 nm and an emission of 422 nm. The images contained within Figure 1, Figure 2, Figure 3, and Figure S1 are from a single animal but representative of the results observed for the treatment group or time point indicated.

## Supporting Information

Figure S1 **Schematic representation of parent miR499 construct.** The structure of the engineered miR499 pre-miRNA sequence includes the antisense target sequence (light purple), the loop sequence (light blue) and the sense sequence with a two-nucleotide deletion (Δ2nt, orange). Vector map of pcDNA6.2-GW/EmGFP miR from Invitrogen. Promoters are purple arrows, origins of replication (ori) are blue arrows, polyadenylation (polyA) signals are yellow bars and antibiotic resistance cassettes are red bars. EmGFP, emerald green fluorescent protein; CMV, cytomegalovirus; TK, thymidine kinase; EM7, EM7 promoter; SV40, simian virus 40; pUC, plasmid “University of California”.
(TIF)

Figure S2 **Histological assessment of neuronal health following rAAV1/2 EmGFP miR499 infusion.** **A**. Colocalisation of EmGFP transgene expression (green) and NeuN-like immunoreactivity (blue) in the absence of relaxin-3 immunoreactivity (red) in the nucleus incertus. Scale bar indicates 50 µm. B. Colocalisation of EmGFP transgene expression (green) and Nissl substance. Scale bar indicates 200 µm.
(TIF)

Figure S3 **Validation of internal control gene and primer efficiency for relaxin-3 and RXFP3 qRT-PCR.** For the two potential internal control genes, β actin (Α) and GAPDH (B), the mean ± SEM of the 2^−CT^ values determined for four animals in each group using quantitative reverse transcription PCR were plotted. *indicates a significant difference between rAAV1/2 EmGFP miR499 and the group indicated, as determined by a two-way repeated-measures ANOVA with Holm-Sidak post-hoc analysis with significance set to p<0.05. C and D. The efficiency of amplification of target gene and the internal control gene was examined. Serial dilutions of pooled control cDNA were amplified using qPCR and the gene-specific primers. The average C_T_ from triplicate determinations was plotted for each cDNA dilution. Linear regression analysis was used to determine the lines of best fit. There was no significant difference between the slopes of each line of best fit for relaxin-3 and GAPDH (C. relaxin-3, r^2^ = 0.968; GAPDH, r^2^ = 0.981, F_(1, 10)_ = 1.299, p = 0.281) or RXFP3 and GAPDH (D. RXFP3 r^2^ = 0.991; GAPDH r^2^ = 0.987, F_(1,10)_ = 1.284, p = 0.284).
(TIF)
