# Supplementary figures and images for: Silencing Relaxin-3 in Nucleus Incertus of Adult Rodents: A Viral Vector-based Approach to Investigate Neuropeptide Function
Source: PLoS One. 2012 Aug 2;7(8):e42300. doi: 10.1371/journal.pone.0042300 (PMC3410922; doi:10.1371/journal.pone.0042300)

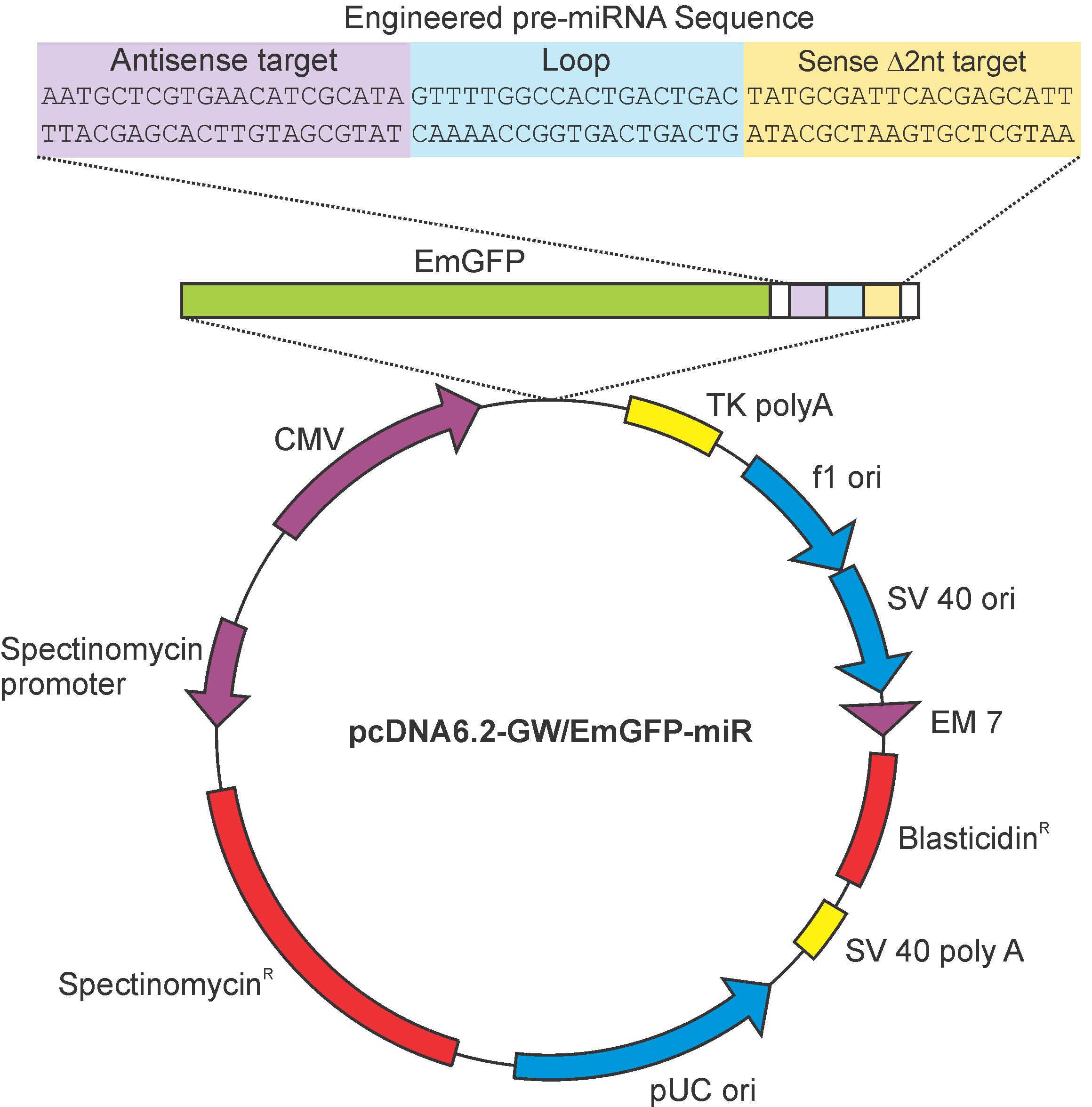

Supplement: Figure S1 — Schematic representation of parent miR499 construct. The structure of the engineered miR499 pre-miRNA sequence includes the antisense target sequence (light purple), the loop sequence (light blue) and the sense sequence with a two-nucleotide deletion (Δ2nt, orange). Vector map of pcDNA6.2-GW/EmGFP miR from Invitrogen. Promoters are purple arrows, origins of replication (ori) are blue arrows, polyadenylation (polyA) signals are yellow bars and antibiotic resistance cassettes are red bars. EmGFP, emerald green fluorescent protein; CMV, cytomegalovirus; TK, thymidine kinase; EM7, EM7 promoter; SV40, simian virus 40; pUC, plasmid “University of California”. (TIF) [file pone.0042300.s001.tif]

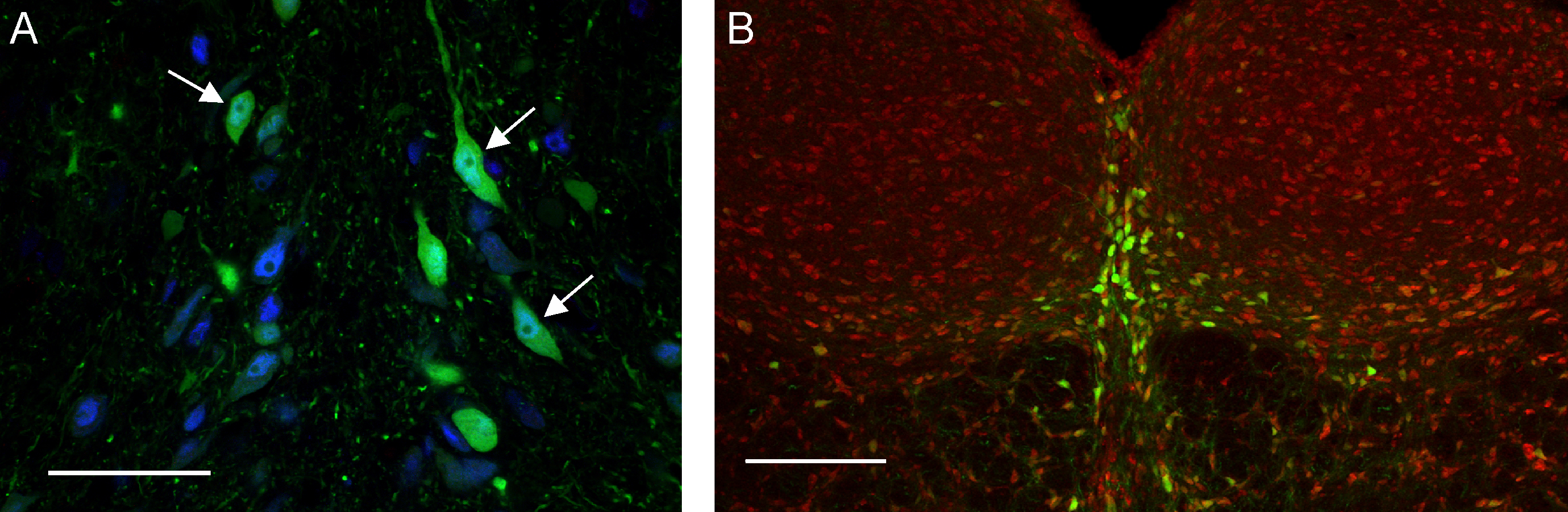

Supplement: Figure S2 — Histological assessment of neuronal health following rAAV1/2 EmGFP miR499 infusion. A. Colocalisation of EmGFP transgene expression (green) and NeuN-like immunoreactivity (blue) in the absence of relaxin-3 immunoreactivity (red) in the nucleus incertus. Scale bar indicates 50 µm. B. Colocalisation of EmGFP transgene expression (green) and Nissl substance. Scale bar indicates 200 µm. (TIF) [file pone.0042300.s002.tif]

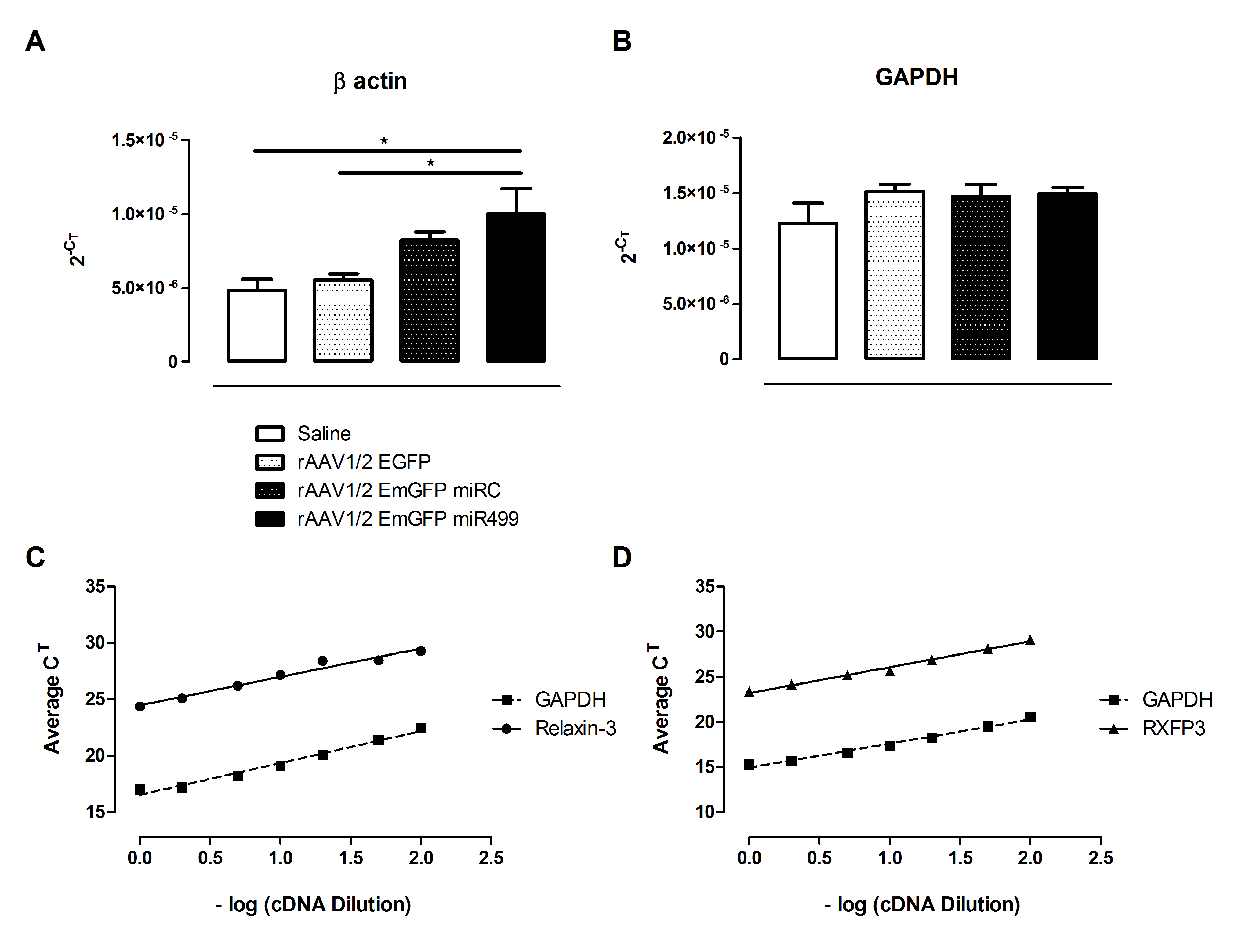

Supplement: Figure S3 — Validation of internal control gene and primer efficiency for relaxin-3 and RXFP3 qRT-PCR. For the two potential internal control genes, β actin (Α) and GAPDH (B), the mean ± SEM of the 2−CT values determined for four animals in each group using quantitative reverse transcription PCR were plotted. *indicates a significant difference between rAAV1/2 EmGFP miR499 and the group indicated, as determined by a two-way repeated-measures ANOVA with Holm-Sidak post-hoc analysis with significance set to p<0.05. C and D. The efficiency of amplification of target gene and the internal control gene was examined. Serial dilutions of pooled control cDNA were amplified using qPCR and the gene-specific primers. The average CT from triplicate determinations was plotted for each cDNA dilution. Linear regression analysis was used to determine the lines of best fit. There was no significant difference between the slopes of each line of best fit for relaxin-3 and GAPDH (C. relaxin-3, r2 = 0.968; GAPDH, r2 = 0.981, F(1, 10) = 1.299, p = 0.281) or RXFP3 and GAPDH (D. RXFP3 r2 = 0.991; GAPDH r2 = 0.987, F(1,10) = 1.284, p = 0.284). (TIF) [file pone.0042300.s003.tif]
